# Cancer, Its Cause and Treatment

**Published:** 1917-07

**Authors:** 


**Cancer, its Cause and Treatment.** By L. DUNCAN BULKLEY, A.M., M.D., Senior Physician, the New York Skin and Cancer Hospital, etc. Pp. 230. New York: Paul B. Hoeber. 1915. Price, $1.50 net.

This book contains six lectures "delivered to practising physicians at the New York Skin and Cancer Hospital." The author's main arguments may be briefly stated thus:—As civilisation increases, cancer becomes more prevalent; amongst uncivilised tribes, such as the aborigines of Australia it is very rare, and it is uncommon amongst vegetarians, and amongst those nations which lead a simple life. The causes of cancer are unknown, but by a process of exclusion we may conclude that the main cause is a faulty metabolism; "malignant disease is but an aberrant action of originally normal body cells." Therefore, the treatment should be medical as well as surgical; correct the faulty metabolism by trying to improve the general health, especially by diminishing the amount of meat that is eaten. He inculcates a "perfect vegetarian diet, which includes even the exclusion of eggs and milk, with food."

He afterwards, however, modifies this statement, and allows the yolk of eggs and some milk. Under this treatment he claims that cases of inoperab e cancer may be improved, and some cases may be cured. He does not condemn removal of the growth; "personally," he writes, "I am occasionally advising this in proper cases" (p. 145). Apparently he believes in local applications, and goes so far as to say: "I have seen the lump vanish under the continued application of the iodide of lead in Hebra's Diachylon Ointment."

It is when he cites figures and actual cases that the falsity of his speculations, and their danger, become apparent. He takes ninety-six cases of so called cancer of the breast, the average duration of the disease in each being one to six years. He owns that in the large majority the diagnosis rested on clinical evidence only, and describes how in four of them the tumour disappeared. In none of these four is there any mention of retraction of the nipple. Three other cases improved under dieting, but in two of these X-rays were used.

As the diagnosis of malignant disease of the breast is obviously uncertain in a large number of cases, without the additional knowledge gained by microscopical examination, the practical surgeon will not consider Dr. Bulkley's contention as proven, and we cannot but regret that arguments should be used which could deter medical men and patients from the only safe course at present known to science—the early and complete removal of malignant tumours of any kind, wherever this is possible.

